# Review of economic evidence in the prevention and early detection of colorectal cancer

**DOI:** 10.1186/2191-1991-3-20

**Published:** 2013-09-12

**Authors:** Kim E Jeong, John A Cairns

**Affiliations:** 1 Department of Health Services Research and Policy, Faculty of Public Health and Policy, London School of Hygiene and Tropical Medicine, 15-17 Tavistock Place, London WC1H 9SH, United Kingdom

**Keywords:** Colorectal cancer, Screening, Polyp, Adenoma, Cost-effectiveness, Cost-utility

## Abstract

This paper aims to systematically review the cost-effectiveness evidence, and to provide a critical appraisal of the methods used in the model-based economic evaluation of CRC screening and subsequent surveillance. A search strategy was developed to capture relevant evidence published 1999-November 2012. Databases searched were MEDLINE, EMBASE, National Health Service Economic Evaluation (NHS EED), EconLit, and HTA. Full economic evaluations that considered costs and health outcomes of relevant intervention were included. Sixty-eight studies which used either cohort simulation or individual-level simulation were included. Follow-up strategies were mostly embedded in the screening model. Approximately 195 comparisons were made across different modalities; however, strategies modelled were often simplified due to insufficient evidence and comparators chosen insufficiently reflected current practice/recommendations. Studies used up-to-date evidence on the diagnostic test performance combined with outdated information on CRC treatments. Quality of life relating to follow-up surveillance is rare. Quality of life relating to CRC disease states was largely taken from a single study. Some studies omitted to say how identified adenomas or CRC were managed. Besides deterministic sensitivity analysis, probabilistic sensitivity analysis (PSA) was undertaken in some studies, but the distributions used for PSA were rarely reported or justified. The cost-effectiveness of follow-up strategies among people with confirmed adenomas are warranted in aiding evidence-informed decision making in response to the rapidly evolving technologies and rising expectations.

## Introduction

Colorectal polyps are small benign growths in the inner layer of the colon and rectum that can be either pre-cancerous or non-precancerous. Neoplastic colorectal polyps, known as adenomas, can be further divided into non-advanced and advanced dependent on the size, degree of villous features, or grade of dysplasia [1,2]. The number and size of adenomas are positively related to the risk of developing colorectal cancer (CRC) over 10 years or longer [1,3,4]. Evidence suggests that early detection and removal of colorectal adenomas (polypectomy) reduces the risk of developing CRC [4].

Several screening modalities are currently used in different sequences and with different intervals ranging from stool tests, barium enema (BE), colonoscopy (COL), sigmoidoscopy (SIG) to computerised tomography colonography (CTC). Each screening modality has particular benefits and potential harms. Despite the absence of sufficient evidence for or against specific CRC screening modalities, CRC screening has been implemented in many countries [5]–[7]. Rapidly evolving technologies and increasing expectations from healthcare users tend to exceed financial affordability and health policy responses in many countries. Guidance is required regarding choice and order of modalities, and appropriate intervals, in order to minimise potential harms and maximise benefits among the eligible population groups. This paper systematically reviews the cost-effectiveness evidence and provides a critical appraisal of methods used in the model-based economic evaluation of CRC screening and subsequent surveillance.

## Review

### Methods

A search strategy was developed (Additional file 1). Databases searched were National Health Service Economic Evaluation Database (NHS EED), EconLit, MEDLINE, EMBASE, and HTA and limited to studies published January 1999 to November 2012. An initial search using the search term ‘surveillance’ was extended to ‘screening’ because of the rarity of published cost-effectiveness analysis of follow-up strategies in the topic area, and also due to terminologies being used interchangeably in the published literature. Key terms used in the search were colonoscopy, surveillance, screening, adenoma, colorectal cancer. Economic filters were used when searching for economic evidence on generalist databases, such as MEDLINE. Simplified searches without economic search filters were performed when searching the economics specific databases [8].

Full economic evaluations that considered costs and health outcomes of relevant types of intervention with outcomes expressed in cost per quality-adjusted life-year (QALY); or cost per life-year gained were included. Studies published pre-1999 [9,10] were reviewed when they were used in the appraisal of newly introduced technologies. Sixty-eight studies were critically appraised by two reviewers using a set of criteria [11]. Further details are described in additional file 2 and additional file 3, and the included studies are summarised in additional file 4.

Findings from selected studies are discussed in the following section.

### Findings

**Figure 1:**
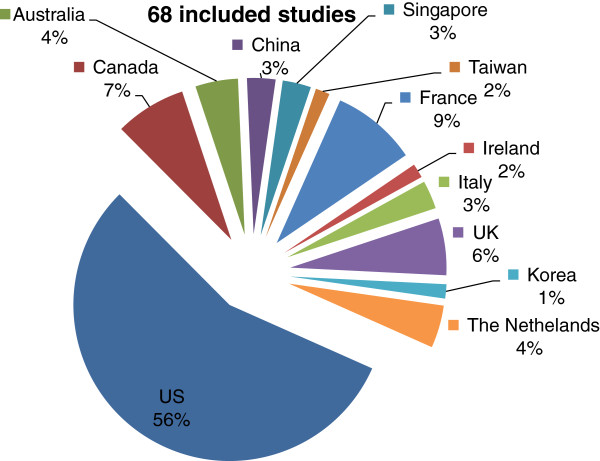
Included studies.

Economic models for surveillance programmes targeting people with a high risk of developing CRC were nested in the main screening model(s) in a number of occasions. The country of origin of the included studies is presented in Figure 1.

#### Modelling methods

Two modelling methods have been used; cohort simulation and individual-level simulation. Some studies provided a limited description of the model [9,12]–[15], others were marginal analyses of cost and benefits derived from published studies that were applied directly to the US population [16].

Computational complexity of the models ranged from a simple decision tree [17]–[24] to a Markov model [10,25]–[57] to capture key aspects of natural history of CRC. Most studies modelled the adenoma-carcinoma sequence over time. Threshold analysis was performed in some studies to investigate the optimal cut-off level for diagnostic tests or optimal reimbursement strategy for a new technology [58].

Individual-level simulation models [58]–[69] have been based on three micro simulation models: Micro Simulation Screening Analysis (MISCAN), Simulated Model of Colorectal Cancer (SimCRC), and the Colorectal Cancer Simulated Population model for Incidence and Natural History (CRC-SPIN). These were independently developed within the National Cancer Institute-funded Cancer Intervention and Surveillance modelling Network (CISNET) consortium. The natural history of CRC in these models was calibrated to autopsy studies and to Surveillance, Epidemiology, and End Results (SEER) Program data for the pre-screening era (1975–1979) [60]. CISNET models subsequently led to a number of secondary analyses [36,66]–[69].

Initiation of CRC screening and subsequent follow-up was mostly around 50 to 60 years of age, while the timing of cessation of screening or surveillance varied. In some surveillance models, people remained in the surveillance programme until the end of the simulation [60,66]. As a result, the surveillance costs would have been overestimated.

#### Population considered

People at average risk were the main focus in most studies, with follow-up surveillance nested in the screening model. For people with positive FOBT results COL was commonly used as a confirmatory test [21,53,68,70]. The importance of follow-up surveillance of individuals at high risk of developing CRC has been recognised in recent years. For example, people with newly diagnosed adenomas were considered in a follow-up strategy using COL compared with no follow-up [44], and people with asymptomatic polyps were followed-up using CTC compared with immediate referral for COL with polypectomy [22,23].

#### Screening modalities considered

The main interventions chosen for modelling were stool tests, COL, SIG progressed to CTC either alone or combined with another modality. CTC was often compared with existing technologies that have emerged in the recent years. Evidence and recommendations on the use of BE remain inconsistent thus BE was considered as one of the current modalities in some studies [20,27,51,56] but excluded in others [47,52].

Stool-based tests, including guiaic FOBT (gFOBT), immunochemical FOBT (iFOBT) and stool DNA tests, were used for mass screening of those at average risk of developing CRC compared with no screening [38,51,71,72]. COL was the common test for the follow-up of detected adenomas/polyps and positive test results from initial screening tests. Unlike COL, SIG provides visualised examination of the left side of the bowel depending on the length of endoscopy and the depth of insertion with no sedation [73]. Narrow-band imaging (NBI) is one of latest technologies with in-vivo histology function compared with conventional white light COL, in which removed adenomas from COL (polypectomy) would be analysed in the lab [29].

**Figure 2:**
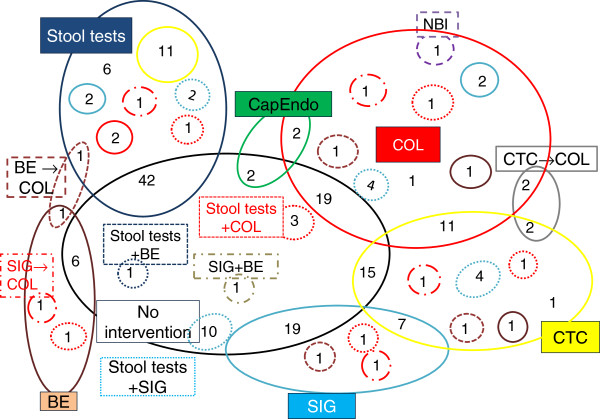
**At a glance.** Barium enema (BE) (brown solid) and colonoscopy (BE + COL) (brown dotted), Capsule endoscopy (CapEndo) (green solid), Computerised-tomography colonography (CTC) (grey solid), Computerised-tomography colonography followed by colonoscopy (CTC**→**COL), Colonoscopy (COL) (red solid), Narrow-band imaging (NBI) (purple dotted), No intervention (black solid). Sigmoidoscopy (SIG) (turquoise solid). Sigmoidoscopy combined with barium enema (SIG + BE) (olive green dashed). Stool tests (blue solid). Stool tests combined with BE (stool tests + BE) (brown solide). Stool tests combined with COL (stool tests + COL) (red dotted), Stool tests combined with SIG (stool tests + SIG) (turquoise dotted), ‘+’ combination of tests,‘**→**’ sequence of test.

Approximately 195 comparisons have been made across the 68 studies (simplifying considerations of the sequence of tests and excluding the interval of screening and follow-up strategies) (Figure 2). This can be partly explained by differences in clinical practice between countries/settings dependent on the structure of health service delivery and reimbursement rules, as well as resource availability. Effectiveness and cost-effectiveness evidence relating to the combination of different tests or their sequence in CRC screening and follow-up was sparse. Stool-based tests were aggregated for simplicity. Each modality is coded using a different colour and shape outline. Numbers shared between circles or within a circle represent the number of comparisons across the studies. For example, NBI was compared with COL once; two comparisons were made of CTC followed by COL and CTC alone.

Threshold analyses at various costs and sensitivity of CTC in detecting polyps were presented in comparison with existing modalities among an average risk population [59,66,69]. Some studies found CTC, with or without a threshold strategy for the size of polyps, would be cost-effective, while others found COL or iFOBT to be cost-effective. This depended on where CTC was used in the screening pathway either primary screening or secondary follow-up test. Cost-effectiveness of CTC was examined in recent years with an improved understanding of the test performance and indications among people with asymptomatic polyps or with a positive result from FOBT [21]–[23]. A definitive follow-up interval using CTC has not been empirically established, thus modelled intervals of CTC strategy varied from every 5 years to 10 years among average risk population, or every 3 years among asymptomatic people with small polyps (6–9 mm) [26,37,49,55].

The potential harm of CTC was rarely considered, although exposure to radiation from CTC every 3 or 5 years was reported to be low [49]. CTC was considered as a primary screening test in an average population compared with FOBT [25], COL [49], SIG [34,60,69]. No studies have considered the costs and consequences of extra colonic findings from CTC.

CTC was not cost-effective as a follow-up test for individuals with positive results from stool tests when compared with COL [24,55]. CTC was relatively cost-effective or cost-saving among people with polyps 6–9 mm [22,23] (Table 1).

**Table 1:** CTC as a follow-up test

	**Population**	**Interventions**	**Sensitivity (Se,%) Specificity (Sp,%) [ranges]**	**Participation rate: initial (I) repeated (R) [Ranges assessed in sensitivity analysis]**	**Reported outcomes**
Pickhardt (2007)	People with small polyps (6–9 mm) detected at CTC screening	CTC with or without polyp size reporting threshold (6-mm) vs COL + polypectomy FSIG No screening	(<=5 mm polyps,6-9 mm, > = 10 mm, CRC) CTC Se (48%, 70%, 85%, 95%) Sp 86% COL Se(80%, 85%, 90%, 95%) Sp 90% FSIG (45%, 45%, 60-65%, 90%)	I 65% [1–100] R 80% [1–100]	Compared with No screening; $4361per LYG (CTC with a 6-mm threshold), $7138 per LYG (CTC with no threshold), $7407 per LYG (FSIG), $9180 per LYG (COL).
					Compared with COL, CTC with a 6-mm threshold resulted in a 77.6% reduction in invasive endoscopic procedures and 1112 fewer reported COL-related complications from perforation or bleeding.
					CTC with non-reporting of diminutive lesions was found to be the most cost-effective and safest screening option evaluated.
Pickhardt (2008a)	60 years old asymptomatic polyps; diminutive (≤5 mm), small (6-9 mm), large (≥10 mm)	CTC then COL with polypectomy vs CTC only	polyps (≤5 mm, ≥6 mm, ≥10 mm,) CTC Se (48%, 89%, 94%) CTC Sp (80%, 8%, 96%)	100% (assumption)	Estimated 10Y CRC risk for unresected diminutive (0.08%), small (0.7%) and large polyps (15.7%). ICER of removing all diminutive polyps was $465,407/LYG, and small CTC-detected polyps $59,015 per LYG. Polypectomy for large CTC-detected polyps yielded a cost-saving of $151 per person screened.
Pickhardt (2008b)	60 years old asymptomatic individuals with small polyps (6- to 9-mm) detected at CTC screening	3-yearly CTC surveillance vs Immediate polypectomy	CTC Se( polyps 6-9 mm) 89%, Sp 80% COL Se( 6-9 mm polyps) 85%, Sp 100%	Not stated	Without any intervention, the estimated 5-year CRC death rate from 6- to 9-mm polyps in this concentrated cohort was 0.08%, which is a sevenfold decrease over the 0.56% CRC risk for the general unselected screening population. The death rate was further reduced to 0.03% with the CTC surveillance strategy and to 0.02% with immediate colonoscopy referral. However, for each additional cancer-related death prevented with immediate polypectomy versus CTC follow-up, 9,977 COL referrals would be needed, resulting in 10 additional perforations and an incremental CE ratio of $372,853.
Walleser (2007)	Individuals with a positive FOBT	CTC vs COL	Se,% (CRC-polyps ≥10 mm - polyps 6-9 mm) CTC Se (89 [70–98]-63 [59–85] - 51 [41–60]) Sp CTC lesions ≥6 mm 90% [88–92] COL Se (96[80–100]-95[90–98]-99[95–100]) Sp COL lesions ≥6 mm 99.6[99.2-100]	Not stated	Australian dollars/LYG
					CTC is less effective and more costly than COL; if CTC was more sensitive than COL, CTC was more effective, at higher cost.

COL (colonoscopy); CRC (colorectal cancer); CTC (computerised tomography colonography); FOBT (fecal occult blood test); FSIG (flexible sigmoidocsopy); LYG (life-years gained).

The frequency and interval of modelled strategies were restricted and simplified compared with day-to-day clinical practice and current guidance/recommendations. This could misrepresent the cost-effectiveness of CTC and other screening modalities [74].

#### Management of polyps/adenomas and CRC

Follow-up was modelled for those with positive results from stool-based tests or polyps detected using endoscopy-based tests or image-based tests. For confirmed polyps, the interval and the degree of complexity of follow-up strategies varied greatly from simple COL at 3–10 years after initial polypectomy to multiple strategies based on the current recommended guidelines [9,57,75]. Follow-up was nested within a Markov model [20,32,38,39] or a discrete event simulation [25,65], or not modelled [37]. Crudely simplified follow-up strategies were considered with assumptions that departed from the real-world, for example, 100% compliance or a common compliance rate at any screening round [9,22,58,61,62]. Cost-effectiveness was generally recognised to depend on compliance with screening, however, one study suggested that high compliance rates were not necessary to achieve cost-effectiveness [19].

Detected polyps were grouped into a single state or two or three depending on number and size of polyps found at baseline COL [30,42,65,76]. Modelled disease states of CRC were mainly local, regional or distant (disseminated) (CRC or Dukes’ stages A to D). In some studies a single CRC disease state was used with an average lifetime treatment cost predicted or estimated thus the results failed to predict benefits of early detection and prevention of CRC [14,19,50]. More recently the costs of CRC stage-specific treatment were modelled including combination and/or sequence of treatments [26,52,70,75]. Costs of CRC treatment were not stated, or were crudely simplified as lifetime costs [50], or directly lifted from previous publications without adjusting to the current year [17,42]. Given the primary goal of screening is prevention and early detection of disease, it is crucial to capture not only the initial years of screening [71] but also the longer term benefits accrued over a lifetime. Any differences in the CRC treatment costs as a result of prevention or early detection of CRC were not distinguished in the model.

#### Input parameters

Since direct evidence on the natural history of CRC is lacking, input parameters were taken from multiple sources ranging from epidemiological studies, hospital records, disease registries and expert opinion.

Papers emphasised the improved test performance of their chosen modalities (and their effectiveness and cost-effectiveness) but often combined more recent information on test performance with existing, outdated information on resource use. For example, the cost-effectiveness of CRC screening with CTC was presented using a single CRC treatment cost taken from a previous study [48] and costs per test from 1998 [42]. COL related complications were modelled in terms of costs. Test performance of CTC varied in the studies from 33% to 100% depending on the size of polyp [22,23,42] (Table 1). In the absence of sensitivity and specificity data for new technologies test performance similar to existing tests was assumed [49]. Quality of life relating to CRC was repeatedly taken from a single study [76] for over a decade [26,45,65,75]. More recently, EQ-5D values of cancer-free and cancer states have been estimated from a national survey [70].

#### Handling uncertainties and model validation

Key assumptions were mainly examined using deterministic sensitivity analyses of the adenoma-carcinoma sequence, CRC prevalence rate, test performance, and compliance rate. In addition, threshold analyses, and scenario analyses were performed to address different types of uncertainty [59,66,69]. However, test performance of screening modalities was not subject to sensitivity analysis in some studies [27,77]. Sensitivity analyses in most cases confirmed the base case finding. Besides uncertainty from sampling variation in the general population, synthesising evidence from multiple sources in order to estimate cost-effectiveness adds another layer of uncertainty. Probabilistic sensitivity analysis (PSA) was performed considering the uncertainty surrounding all parameters simultaneously [13,33,44,55,65] complementing the deterministic sensitivity analyses. The distributions used for PSA were reported in only two studies, although no justification was given for choosing these distributions [25,45]. Uncertainties surrounding input parameters were addressed using appropriate types of sensitivity analyses in some studies, thus improving credibility and robustness of the reported results. For example, a number of scenario analyses were considered in which different adherence rates and lower subsequent adherence rates were applied across strategies [75]. Results were sensitive to costs, but sometimes cost data were not considered in sensitivity analyses [42]. Other studies did not address limitations related to their assumptions [12,15,56]. Methods for economic evaluation have been consolidated further over time, and authors have accordingly explored uncertainty to a greater extent in recent publications.

Validation of models is desirable in order to minimise errors and improve study credibility, and consistency with methodological guides [78]. Model results were not validated in early publications because no data set was available [10,61,62]. An extensive ‘debudding exercise’ and the review of model structure by independent clinicians were reported as internal validation [75]. Validation of models was performed by comparing model simulation results with actual data sets [17,28,40,42]–[44,54,77] or by calibration against published studies [32,39,59].

Validation results showed overestimated efficacy for polypectomy [29], underestimated prevalence of adenoma compared with an existing study [37], or significantly different CRC incidence compared with a recent publication [23], slightly underestimated CRC mortality compared with existing studies [45], or model’s prediction of CRC incidence reduction was consistent with available data [47].

### Discussion

Evidence on the natural history of CRC is limited. The studies identified were predominantly model-based economic evaluations; because no single trial could provide the large sample and long-term follow-up data required to compare screening strategies with differing screening intervals, and sequences/combinations of tests. The assumed constant risks of individuals developing CRC would have under- or over-estimated CRC incidence and subsequent resource use for its treatment.

In clinical practice, a sequence of the same or different tests is performed in CRC screening. Compared to current practice, the modalities modelled were limited and the adenoma-carcinoma sequence was crudely simplified. As a consequence of rapidly evolving technology and the quite poor evidence base regarding natural history, costs, and health outcomes, many evaluations have been of limited value in informing routine clinical practice.

It is vital to know which test(s) should be considered first in which population, or in what combination or sequence, in order to maximise health benefit considering best available effectiveness and cost-effectiveness evidence in the prevention and early detection of CRC. For example, CTC appeared to be cost-ineffective as a primary screening modality compared with other tests among average risk population, but potentially could be cost-effective when used as a follow-up test in a selected population in a pathway. A pathway for CRC including screening, follow-up surveillance and treatment for CRC would provide a bigger picture compared with studies that provide a snapshot view [79]. Given the computational complexity and additional data required for a pathway model, a balance must be struck between transparency and flexibility when choosing the modelling approach in each context.

The studies often omitted to say (or simplified) how identified adenomas or CRC were to be managed or treated. CRC screening and follow-up tests aim to detect early CRC or prevent CRC, thus the consequent costs and health benefits should be accounted for in the model. The improved test performance of newer modalities was captured, but their downstream effects for screening/follow-up were dated. Current or existing guidance on the cost-effectiveness analysis of CRC treatments should be linked to the diagnostic tests when estimating cost-effectiveness of CRC screening and follow-up strategies. This is because the cost-effectiveness of a diagnostic strategy depends in part on the consequences for subsequent treatment. Furthermore, for the cost-effectiveness of a new treatment evidence tends to be generated through randomised clinical trials. However, input parameters for quality of life have suffered from selection bias because searches for data have not been conducted as systematically, and values generally have come from observational studies. Efforts should be made to have up-to-date input parameters for down-stream effects in order to estimate cost-effectiveness of new modalities with less bias and uncertainty.

Test performance and compliance rates will vary between screening round and subsequent follow-up testing. Such variations were crudely simplified by assuming a fixed test performance and a constant compliance rate, and were explored in a deterministic sensitivity analysis in most studies. Further studies varying test performance and compliance rates at each screening round dependent on different tests are recommended.

Extra colonic findings from CTC will influence average screening costs and the subsequent health outcomes, and therefore should be considered in order to estimate the relevant costs and health outcomes of CTC strategies.

The time period during which the cancer is asymptomatic but detectable by the screening test or the time by which the CRC was diagnosed through screening were insufficiently modelled and explored in sensitivity analyses. Assumptions are necessary when constructing a model and uncertainties are introduced at various stages, for example, multiple sources of key parameters to populate the model (parameter uncertainty), and the choice of health states (structural uncertainty). Sensitivity analyses of carefully chosen aspects of uncertainties can increase confidence in or question results. Due to the limited evidence on the natural history of the adenoma-carcinoma sequence, key assumptions are required, however, the subsequent structural uncertainty was not fully explored in most studies. Alternative choices of health states or care pathways should be explored using different scenario analyses. Parameter uncertainty was not fully explored, although uncertainties around mean health and mean cost were explored to a degree. Cost data were rarely explored in PSA, and when they were the distributions were poorly justified.

Cost-effectiveness of follow-up strategies and the inter-relation between CRC screening and follow-up programmes need further study. In addition, other factors, such as healthcare financing and delivery of health service, should also be considered because a modality can be cost-effective in a specific setting, however, this does not guarantee cost-effectiveness in a different setting.

CRC screening and follow-up tests can be invasive with unintended consequences, such as perforation and bleeding, and also involve pre-procedural preparation and post-procedure rest. These impacts on quality of life, have been under-studied and under-reported in most studies. Quality of life data in relation to CRC and colorectal adenoma are very limited, and for over a decade were largely based on a single study [80]. It is imperative to establish a better understanding of the impact on quality of life of CRC screening and follow-up in people with adenomas and CRC.

## Conclusion

Despite many cost-effectiveness analyses having been published important aspects remains under-researched, including the consideration of downstream effects (such as management of adenoma and CRC) linked to appropriate screening or follow-up tests. It is important to assess the cost-effectiveness of different combinations or sequences of follow-up strategies for those with positive results and identified adenomas from mass screening. Information generated will serve as a key link between a mass CRC screening programme and the most appropriate follow-up tests and relevant treatments, and will also aid decision makers to introduce appropriate guidance/policy, and will guide clinical practitioners as to clinically effective and cost-effective follow-up strategies to offer appropriate individuals. Therefore, cost-effectiveness analysis of follow-up tests for people with confirmed adenomas is warranted.

## Abbreviations

BE: Air-contrast barium enema; CapEndo: Capsule endoscope; COL: Colonoscopy; CRC: Colorectal cancer; CTC: Computerised tomography colonography; FOBT: Fecal occult blood test; SIG: Sigmoidoscopy; gFOBT: Guaiac fecal occult blood test; iFOBT: Immunological fecal occult blood test; NBI: Narrow-band imaging.

## Competing interests

Both authors declare that they have no competing interests.

## Authors’ contributions

KJ and JC were involved in all stages of literature search, sifting, and review of studies, revising manuscript critically for important intellectual content. First draft manuscript was written by KJ. Both authors read and approved the final manuscript.

## Supplementary Material

Additional file 1 Search strategy for MEDLINE. Click here for file

Additional file 2 Selection criteria. Click here for file

Additional file 3 Included/excluded studies. Click here for file

Additional file 4 Summary of included studies. Click here for file
