# Supplementary material for: Review of economic evidence in the prevention and early detection of colorectal cancer
Source: Health Econ Rev. 2013 Sep 12;3:20. doi: 10.1186/2191-1991-3-20 (PMC3847082; doi:10.1186/2191-1991-3-20)
Supplement: Additional file 1 — Search strategy for MEDLINE. [file 2191-1991-3-20-S1.docx]

Additional file 1. Search strategy for MEDLINE

Database(s): **Ovid MEDLINE(R) In-Process & Other Non-Indexed Citations and Ovid MEDLINE(R)** 1946 to Present
Search Strategy:

| **#** | **Searches** | **Results** |
| --- | --- | --- |
| 1 | ((colorectal or colon$ or rectum or rectal) adj2 (cancer$ or tumour$ or tumor$ or neoplasm$ or carcinoma$ or adenoma$ or polyp$)).ti,ab. | 115391 |
| 2 | Colonoscopy/ | 16294 |
| 3 | colonoscop$.ti,ab. | 16760 |
| 4 | sigmoidoscop$.ti,ab. | 3592 |
| 5 | exp Mass Screening/ | 93902 |
| 6 | exp Population Surveillance/ | 46155 |
| 7 | Diagnostic tests, routine/ | 6251 |
| 8 | screen$.ti,ab. | 415503 |
| 9 | 2 or 3 or 4 | 26014 |
| 10 | 5 or 6 or 7 or 8 | 495330 |
| 11 | 1 and 9 and 10 | 4432 |
| 12 | limit 11 to yr="1999 -Current" | 3671 |
| 13 | Economics/ or exp "Costs and Cost Analysis"/ or Economics, Dental/ or exp Economics, Hospital/ or exp Economics, Medical/ or Economics, Nursing/ or Economics, Pharmaceutical/ or Budgets/ or exp Models, Economic/ or Markov Chains/ or Monte Carlo Method/ or Decision Trees/ | 256934 |
| 14 | (Economic* or cost or costs or costly or costing or costed or price or prices or pricing or pharmacoeconomic$ or pharmaco economic$ or budget*).ti,ab. | 420310 |
| 15 | ((monte adj carlo) or markov or (decision adj2 (tree$ or analys$))).ti,ab. | 40324 |
| 16 | Quality-Adjusted Life Years/ | 6140 |
| 17 | (quality adjusted life or qaly*).ti,ab. | 6297 |
| 18 | (disability adjusted life or daly).ti,ab. | 1231 |
| 19 | (value adj2 (money or monetary)).ti,ab. | 1173 |
| 20 | 13 or 14 or 15 or 16 or 17 or 18 or 19 | 580430 |
| 21 | 12 and 20 | 610 |
