# Supplementary material for: Review of economic evidence in the prevention and early detection of colorectal cancer
Source: Health Econ Rev. 2013 Sep 12;3:20. doi: 10.1186/2191-1991-3-20 (PMC3847082; doi:10.1186/2191-1991-3-20)
Supplement: Additional file 2 — Selection criteria. [file 2191-1991-3-20-S2.docx]

Additional file 2. Selection criteria

Full economic evaluations that considered costs and health outcomes of relevant types of intervention with outcomes expressed in cost per quality-adjusted life-year (QALY); or cost per life-year gained. Burden of disease studies or non-comparative costing studies were excluded.

Any studies which did not assess costs and related health outcomes in line with the research questions were excluded. The population considered was adults with confirmed colorectal adenoma/polyp who are otherwise healthy with no personal or familial history of CRC. Follow-up strategies and screening strategies based on the best available evidence on the screening modalities were considered including current practice and no intervention. Clinical investigation or therapeutic interventions for suspected CRC or conditions other than colorectal adenoma/polyp were not considered.

Title and abstracts were screened and papers that did not meet the inclusion criteria were excluded. Full text was acquired for the remaining studies. When studies presented insufficient detail (for example, no abstract available) full-text was reviewed. All abstracts were screened, and any disagreements were resolved by discussion between two reviewers.

Data were extracted on author(s), year of publication, modelling approach, screening modalities, population groups, perspective of analysis and analytic horizon, effectiveness data sources, outcome measures, cost elements, cost data sources, year of costing reported, adjustment for inflation, discount rate, baseline results, variables used in the results and sensitivity analyses, reported limitations, reported model validation, and reported conclusion. In addition, modelled strategies for follow-up of positive results from first-line screening and follow-up of adenoma/polyp and CRC treatments were reviewed.
