# Supplementary material for: Review of economic evidence in the prevention and early detection of colorectal cancer
Source: Health Econ Rev. 2013 Sep 12;3:20. doi: 10.1186/2191-1991-3-20 (PMC3847082; doi:10.1186/2191-1991-3-20)
Supplement: Additional file 3 — Included/excluded studies. [file 2191-1991-3-20-S3.docx]

Additional file 3. Included/excluded studies

Included studies

1. Chauvin, P., J.M. Josselin, and D. Heresbach, Incremental net benefit and acceptability of alternative health policies: A case study of mass screening for colorectal cancer. European Journal of Health Economics, 2012. 13(3): p. 237-250.

2. Dan, Y.Y., et al., Screening based on risk for colorectal cancer is the most cost-effective approach. Clinical Gastroenterology & Hepatology, 2012. 10(3): p. 266-71.e1-6.

3. Di Bidino, R., et al., Impact of technology overlapping: a case study on colorectal cancer screening. Technology & Health Care, 2010. 18(4-5): p. 303-15.

4. Eddy, D.M., Screening for colorectal cancer. American Journal of Physicians, 1990. 113: p. 373-384.

5. Flanagan, W.M., et al., Potential impact of population-based colorectal cancer screening in Canada. Chronic Diseases in Canada, 2003. 24(4): p. 81-8.

6. Frazier, A.L., et al., Cost-effectiveness of screening for colorectal cancer in the general population. JAMA, 2000. 284(15): p. 1954-61.

7. Hassan, C., et al., Value-of-information analysis to guide future research in colorectal cancer screening. Radiology, 2009b. 253(3): p. 745-752.

8. Hassan, C., et al., Cost-effectiveness of early one-year colonoscopy surveillance after polypectomy. Diseases of the Colon & Rectum, 2009a. 52(5): p. 964-71; discussion 971.

9. Hassan, C., P.J. Pickhardt, and D.K. Rex, A resect and discard strategy would improve cost-effectiveness of colorectal cancer screening. Clinical Gastroenterology & Hepatology, 2010. 8(10): p. 865-9, 869.e1-3.

10. Hassan, C., et al., Colon cancer prevention in Italy: cost-effectiveness analysis with CT colonography and endoscopy. Digestive & Liver Disease, 2007. 39(3): p. 242-50.

11. Hassan, C., et al., Cost-effectiveness of capsule endoscopy in screening for colorectal cancer. Endoscopy, 2008. 40(5): p. 414-21.

12. Heitman, S.J., et al., Colorectal cancer screening for average-risk North Americans: an economic evaluation. PLoS Medicine / Public Library of Science, 2010. 7(11): p. e1000370.

13. Heitman, S.J., et al., Cost-effectiveness of computerized tomographic colonography versus colonoscopy for colorectal cancer screening. CMAJ Canadian Medical Association Journal, 2005. 173(8): p. 877-81.

14. Helm, J.F., et al., Effectiveness and economic impact of screening for colorectal cancer by mass fecal occult blood testing. American Journal of Gastroenterology, 2000. 95(11): p. 3250-8.

15. Heresbach, D., et al., Cost-effectiveness of colorectal cancer screening with computed tomography colonography or fecal blood tests. European Journal of Gastroenterology & Hepatology, 2010b. 22(11): p. 1372-9.

16. Heresbach, D., et al., Cost-effectiveness of colorectal cancer screening with computed tomography colonography according to a polyp size threshold for polypectomy. European Journal of Gastroenterology & Hepatology, 2010a. 22(6): p. 716-23.

17. Ho, C., et al., Computed tomographic colonography for colorectal cancer screening in an average risk population: Systematic review and economic evaluation (Structured abstract). Ottawa: Canadian Agency for Drugs and Technologies in Health (CADTH), 2008: p. 153.

18. Howard, K., et al., High participation rates are not necessary for cost-effective colorectal cancer screening. Journal of Medical Screening, 2005. 12(2): p. 96-102.

19. Khandker, R.K., et al., A decision model and cost-effectiveness analysis of colorectal cancer screening and surveillance guidelines for average-risk adults. International Journal of Technology Assessment in Health Care, 2000. 16(3): p. 799-810.

20. Knudsen, A.B., et al., Cost-effectiveness of computed tomographic colonography screening for colorectal cancer in the medicare population. Journal of the National Cancer Institute, 2010. 102(16): p. 1238-52.

21. Ladabaum, U. and K.A. Phillips, Colorectal cancer screening differential costs for younger versus older Americans. American Journal of Preventive Medicine, 2006. 30(5): p. 378-84.

22. Ladabaum, U., K. Song, and A.M. Fendrick, Colorectal neoplasia screening with virtual colonoscopy: when, at what cost, and with what national impact? Clinical Gastroenterology & Hepatology, 2004. 2(7): p. 554-63.

23. Lansdorp-Vogelaar, I., et al., Stool DNA testing to screen for colorectal cancer in the Medicare population: a cost-effectiveness analysis. Annals of Internal Medicine, 2010. 153(6): p. 368-77.

24. Lansdorp-Vogelaar, I., et al., At what costs will screening with CT colonography be competitive? A cost-effectiveness approach. International Journal of Cancer, 2009a. 124(5): p. 1161-8.

25. Lansdorp-Vogelaar, I., et al., Individualizing colonoscopy screening by sex and race. Gastrointestinal Endoscopy, 2009b. 70(1): p. 96-108, 108.e1-24.

26. Lee, D., et al., Cost Effectiveness of CT Colonography for UK NHS Colorectal Cancer Screening of Asymptomatic Adults Aged 60-69 Years. Applied Health Economics and Health Policy, 2010. 8(3): p. 141-54.

27. Lejeune, C., et al., Cost-effectiveness analysis of fecal occult blood screening for colorectal cancer. International Journal of Technology Assessment in Health Care, 2004. 20(4): p. 434-9.

28. Lejeune, C., et al., Cost-effectiveness of screening for colorectal cancer in France using a guaiac test versus an immunochemical test. International Journal of Technology Assessment in Health Care, 2010. 26(1): p. 40-7.

29. Loeve, F., et al., The MISCAN-COLON simulation model for the evaluation of colorectal cancer screening. Computers & Biomedical Research, 1999. 32(1): p. 13-33.

30. Loeve, F., et al., Endoscopic colorectal cancer screening: a cost-saving analysis. Journal of the National Cancer Institute, 2000. 92(7): p. 557-63.

31. Macafee, D.A.L., et al., Population screening for colorectal cancer: the implications of an ageing population. British Journal of Cancer, 2008. 99(12): p. 1991-2000.

32. Maciosek, M.V., et al., Colorectal cancer screening: health impact and cost effectiveness. American Journal of Preventive Medicine, 2006. 31(1): p. 80-9.

33. McMahon, P.M., et al., Cost-effectiveness of colorectal cancer screening. Radiology, 2001. 219(1): p. 44-50.

34. Ness, R.M., et al., Cost-utility of one-time colonoscopic screening for colorectal cancer at various ages. American Journal of Gastroenterology, 2000. 95(7): p. 1800-11.

35. O'Leary, B.A., et al., Cost-effectiveness of colorectal cancer screening: comparison of community-based flexible sigmoidoscopy with fecal occult blood testing and colonoscopy. Journal of Gastroenterology & Hepatology, 2004. 19(1): p. 38-47.

36. Parekh, M., A.M. Fendrick, and U. Ladabaum, As tests evolve and costs of cancer care rise: reappraising stool-based screening for colorectal neoplasia. Alimentary pharmacology & therapeutics, 2008. 27(8): p. 697-712.

37. Park, S.M., Y.H. Yun, and S. Kwon, Feasible economic strategies to improve screening compliance for colorectal cancer in Korea. World Journal of Gastroenterology, 2005. 11(11): p. 1587-93.

38. Pickhardt, P.J., et al., Small and diminutive polyps detected at screening CT colonography: a decision analysis for referral to colonoscopy. AJR. American Journal of Roentgenology, 2008a. 190(1): p. 136-44.

39. Pickhardt, P.J., et al., Clinical management of small (6- to 9-mm) polyps detected at screening CT colonography: a cost-effectiveness analysis. AJR. American Journal of Roentgenology, 2008b. 191(5): p. 1509-16.

40. Pickhardt, P.J., et al., Cost-effectiveness of colorectal cancer screening with computed tomography colonography: the impact of not reporting diminutive lesions. Cancer, 2007. 109(11): p. 2213-21.

41. Regge, D., et al., Impact of computer-aided detection on the cost-effectiveness of CT colonography. Radiology, 2009. 250(2): p. 488-497.

42. Saini, S.D., P. Schoenfeld, and S. Vijan, Surveillance colonoscopy is cost-effective for patients with adenomas who are at high risk of colorectal cancer. Gastroenterology, 2010. 138(7): p. 2292-9, 2299.e1.

43. Sharp, L., et al., Cost-effectiveness of population-based screening for colorectal cancer: a comparison of guaiac-based faecal occult blood testing, faecal immunochemical testing and flexible sigmoidoscopy. British Journal of Cancer, 2012. 106(5): p. 805-16.

44. Sobhani, I., et al., Cost-effectiveness of mass screening for colorectal cancer: choice of fecal occult blood test and screening strategy. Diseases of the Colon & Rectum, 2011. 54(7): p. 876-86.

45. Song, K., A.M. Fendrick, and U. Ladabaum, Fecal DNA testing compared with conventional colorectal cancer screening methods: a decision analysis. Gastroenterology, 2004. 126(5): p. 1270-9.

46. Sonnenberg, A. and F. Delco, Cost-effectiveness of a single colonoscopy in screening for colorectal cancer. Archives of Internal Medicine, 2002. 162(2): p. 163-8.

47. Sonnenberg, A., F. Delco, and P. Bauerfeind, Is virtual colonoscopy a cost-effective option to screen for colorectal cancer? American Journal of Gastroenterology, 1999. 94(8): p. 2268-74.

48. Sonnenberg, A., F. Delco, and J.M. Inadomi, Cost-effectiveness of colonoscopy in screening for colorectal cancer. Annals of Internal Medicine, 2000. 133(8): p. 573-84.

49. Stone, C.A., et al., Colorectal cancer screening in Australia: an economic evaluation of a potential biennial screening program using faecal occult blood tests. Australian & New Zealand Journal of Public Health, 2004. 28(3): p. 273-82.

50. Subramanian, S., G. Bobashev, and R.J. Morris, Modeling the cost-effectiveness of colorectal cancer screening: policy guidance based on patient preferences and compliance. Cancer Epidemiology, Biomarkers & Prevention, 2009. 18(7): p. 1971-8.

51. Tappenden, P., et al., Option appraisal of population-based colorectal cancer screening programmes in England. Gut, 2007. 56(5): p. 677-84.

52. Telford, J.J., et al., The cost-effectiveness of screening for colorectal cancer. CMAJ Canadian Medical Association Journal, 2010. 182(12): p. 1307-13.

53. Theuer, C.P., et al., Gender and race/ethnicity affect the cost-effectiveness of colorectal cancer screening. Journal of the National Medical Association, 2006. 98(1): p. 51-7.

54. Theuer, C.P., et al., Racial and ethnic colorectal cancer patterns affect the cost-effectiveness of colorectal cancer screening in the United States. Gastroenterology, 2001. 120(4): p. 848-56.

55. Tsoi, K.K.F., et al., Cost-effectiveness analysis on screening for colorectal neoplasm and management of colorectal cancer in Asia. Alimentary pharmacology & therapeutics, 2008. 28(3): p. 353-63.

56. van Rossum, L.G., et al., Colorectal cancer screening comparing no screening, immunochemical and guaiac fecal occult blood tests: a cost-effectiveness analysis. International Journal of Cancer, 2011. 128(8): p. 1908-17.

57. Vanness, D.J., et al., Comparative economic evaluation of data from the ACRIN national CT colonography trial with three cancer intervention and surveillance modeling network microsimulations. Radiology, 2011. 261(2): p. 487-498.

58. Vijan, S., et al., Which colon cancer screening test? A comparison of costs, effectiveness, and compliance. American Journal of Medicine, 2001. 111(8): p. 593-601.

59. Vijan, S., et al., The cost-effectiveness of CT colonography in screening for colorectal neoplasia. American Journal of Gastroenterology, 2007. 102(2): p. 380-90.

60. Wagner, J.L., Herdman, R. C., Wadhwa, S., Cost effectiveness of colorectal cancer screening in the elderly. Annals of Internal Medicine, 1991. 115(10): p. 8007-817.

61. Walleser, S., et al., What is the value of computered tomography colonography in patients screening positive for fecal occult blood? A systematic review and economic evaluation. Clinical Gastroenterology & Hepatology, 2007. 5(12): p. 1439-46; quiz 1368.

62. Wang, Z.H., Q.Y. Gao, and J.Y. Fang, Repeat colonoscopy every 10 years or single colonoscopy for colorectal neoplasm screening in average-risk Chinese: a cost-effectiveness analysis. Asian Pacific Journal of Cancer Prevention: Apjcp, 2012. 13(5): p. 1761-6.

63. Whyte, S., Reappraisal of the options for colorectal cancer screening in England. Colorectal Disease, 2012. 14(9): p. e547-61.

64. Wilschut, J.A., et al., Fecal occult blood testing when colonoscopy capacity is limited. Journal of the National Cancer Institute, 2011b. 103(23): p. 1741-51.

65. Wilschut, J.A., et al., Cost-effectiveness analysis of a quantitative immunochemical test for colorectal cancer screening. Gastroenterology, 2011a. 141(5): p. 1648-55.e1.

66. Wong, S.S., A.P. Leong, and T.Y. Leong, Cost-effectiveness analysis of colorectal cancer screening strategies in Singapore: a dynamic decision analytic approach. Medinfo, 2004. MEDINFO. 11(Pt 1): p. 104-110.

67. Wu, G.H.-M., et al., Cost-effectiveness analysis of colorectal cancer screening with stool DNA testing in intermediate-incidence countries. BMC Cancer, 2006. 6: p. 136.

68. Zauber, A.G., Cost-effectiveness of colonoscopy. Gastrointestinal Endoscopy Clinics of North America, 2010. 20(4): p. 751-70.

Excluded studies after full-text review

1. Allameh Z, Davari M, Emami MH. Cost-effectiveness analysis of colorectal cancer screening methods in Iran. Archives of Iranian Medicine. 2011 Mar;14(2):110-4. PubMed PMID: 21361717. English.

2. Arnesen RB, Ginnerup-Pedersen B, Poulsen PB, von Benzon E, Adamsen S, Laurberg S, et al. Cost-effectiveness of computed tomographic colonography: a prospective comparison with colonoscopy. Acta Radiol. 2007 Apr;48(3):259-66. PubMed PMID: 17453492. English.

3. Becker F, Nusko G, Welke J, Hahn EG, Mansmann U. Benefit-risk analysis of different risk-related surveillance schedules following colorectal polypectomy. Hepatogastroenterology. 2007 Dec;54(80):2249-58. PubMed PMID: 18265643. English.

4. Berchi C, Bouvier V, Reaud J-M, Launoy G. Cost-effectiveness analysis of two strategies for mass screening for colorectal cancer in France. Health Economics. 2004 Mar;13(3):227-38. PubMed PMID: 14981648. English.

5. Bradley CJ, Lansdorp-Vogelaar I, Yabroff KR, Dahman B, Mariotto A, Feuer EJ, et al. Productivity savings from colorectal cancer prevention and control strategies. American Journal of Preventive Medicine. 2011 Aug;41(2):e5-e14. PubMed PMID: 21767717. Pubmed Central PMCID: NIHMS307125 [Available on 08/01/12] PMC3139918 [Available on 08/01/12]. English.

6. Buset M, Huybrechts M. Economic impact of a colorectal cancer screening programme in Belgium. Acta Gastroenterol Belg. 2005 Apr-Jun;68(2):262-3. PubMed PMID: 16013647. English.

7. Cahill BA. Colorectal cancer. Which test is best? Advance for Nurse Practitioners. 2005 Jan;13(1):71-4. PubMed PMID: 15679298. English.

8. Canadian COfHTA. Economic evaluation of population-based screening for colorectal cancer (Brief record). Ottawa: Canadian Coordinating Office for Health Technology Assessment (CCOHTA). 2002.

9. Chen L-S, Liao C-S, Chang S-H, Lai H-C, Chen TH-H. Cost-effectiveness analysis for determining optimal cut-off of immunochemical faecal occult blood test for population-based colorectal cancer screening (KCIS 16). Journal of Medical Screening. 2007;14(4):191-9. PubMed PMID: 18078564. English.

10. Chiu SYH, Malila N, Yen AMF, Anttila A, Hakama M, Chen HH. Analytical decision model for sample size and effectiveness projections for use in planning a population-based randomized controlled trial of colorectal cancer screening. Journal of Evaluation in Clinical Practice. 2011 Feb;17(1):123-9. PubMed PMID: 20831662. English.

11. Church JM. Colon cancer screening update and management of the malignant polyp. Clin. 2005 Aug;18(3):141-9. PubMed PMID: 20011297. Pubmed Central PMCID: PMC2780100. English.

12. Delco F, Sonnenberg A. At what age should a one-time only colonoscopy for screening of colorectal cancer be performed? Eur J Gastroenterol Hepatol. 1999 Nov;11(11):1319-20. PubMed PMID: 10563547. English.

13. Delco F, Sonnenberg A. Limitations of the faecal occult blood test in screening for colorectal cancer. Ital J Gastroenterol. 1999 Mar;31(2):119-26. PubMed PMID: 10363196. English.

14. Fisher JA, Fikry C, Troxel AB. Cutting cost and increasing access to colorectal cancer screening: another approach to following the guidelines. Cancer Epidemiol Biomarkers Prev. 2006 Jan;15(1):108-13. PubMed PMID: 16434595. English.

15. Frew E, Wolstenholme JL, Whynes DK. Willingness-to-pay for colorectal cancer screening. European Journal of Cancer. 2001 Sep;37(14):1746-51. PubMed PMID: 11549427. English.

16. Grazzini G, Ciatto S, Cislaghi C, Castiglione G, Falcone M, Mantellini P, et al. Cost evaluation in a colorectal cancer screening programme by faecal occult blood test in the District of Florence. Journal of Medical Screening. 2008;15(4):175-81. PubMed PMID: 19106257. English.

17. Griffith S, Kane KY. What is the most cost-effective screening regimen for colon cancer? J. 2001 Jan;50(1):13. PubMed PMID: 11195472. English.

18. Hassan C, Pickhardt PJ, Di Giulio E, Hunink MGM, Zullo A, Nardelli BB. Value-of-information analysis to guide future research in the management of the colorectal malignant polyp. Dis Colon Rectum. 2010 Feb;53(2):135-42. PubMed PMID: 20087087. English.

19. Haug U, Brenner H. A simulation model for colorectal cancer screening: potential of stool tests with various performance characteristics compared with screening colonoscopy. Cancer Epidemiol Biomarkers Prev. 2005 Feb;14(2):422-8. PubMed PMID: 15734968. English.

20. Henry SG, Ness RM, Stiles RA, Shintani AK, Dittus RS. A cost analysis of colonoscopy using microcosting and time-and-motion techniques. Journal of General Internal Medicine. 2007 Oct;22(10):1415-21. PubMed PMID: 17665271. Pubmed Central PMCID: PMC2305858. English.

21. Inadomi JM, Sonnenberg A. The impact of colorectal cancer screening on life expectancy. Gastrointestinal Endoscopy. 2000 May;51(5):517-23. PubMed PMID: 10805834. English.

22. Ladabaum U, Ferrandez A, Lanas A. Cost-effectiveness of colorectal cancer screening in high-risk Spanish patients: use of a validated model to inform public policy. Cancer Epidemiol Biomarkers Prev. 2010 Nov;19(11):2765-76. PubMed PMID: 20810603. Pubmed Central PMCID: NIHMS311762

PMC3159034. English.

23. Lejeune C, Arveux P, Dancourt V, Fagnani F, Bonithon-Kopp C, Faivre J. A simulation model for evaluating the medical and economic outcomes of screening strategies for colorectal cancer. Eur J Cancer Prev. 2003 Feb;12(1):77-84. PubMed PMID: 12548114. English.

24. Li S, Wang H, Hu J, Li N, Liu Y, Wu Z, et al. New immunochemical fecal occult blood test with two-consecutive stool sample testing is a cost-effective approach for colon cancer screening: results of a prospective multicenter study in Chinese patients. International Journal of Cancer. 2006 Jun 15;118(12):3078-83. PubMed PMID: 16425283. English.

25. Lieberman D. Cost-effectiveness of colonoscopy in screening for colorectal cancer; cost-effectiveness of screening colorectal cancer in the general population. Gastrointestinal Endoscopy. 2001 Oct;54(4):537-8. PubMed PMID: 11601425. English.

26. Lucidarme O, Cadi M, Berger G, Taieb J, Poynard T, Grenier P, et al. Cost-effectiveness modeling of colorectal cancer: computed tomography colonography vs colonoscopy or fecal occult blood tests. Eur J Radiol. 2012 Jul;81(7):1413-9. PubMed PMID: 21444171. English.

27. McGrath JS, Ponich TP, Gregor JC. Screening for colorectal cancer: the cost to find an advanced adenoma. American Journal of Gastroenterology. 2002 Nov;97(11):2902-7. PubMed PMID: 12425566. English.

28. Provenzale D. Cost-effectiveness of screening the average-risk population for colorectal cancer. Gastrointestinal Endoscopy Clinics of North America. 2002 Jan;12(1):93-109. PubMed PMID: 11916165. English.

29. Sekiguchi M, Matsuda T, Tamai N, Sakamoto T, Nakajima T, Otake Y, et al. Cost-effectiveness of total colonoscopy in screening of colorectal cancer in Japan. Gastroenterol Res Pract. 2012;2012:728454. PubMed PMID: 22291697. Pubmed Central PMCID: PMC3265074. English.

30. Sharara N, Adam V, Crott R, Barkun AN. The costs of colonoscopy in a Canadian hospital using a microcosting approach. Canadian Journal of Gastroenterology. 2008 Jun;22(6):565-70. PubMed PMID: 18560635. Pubmed Central PMCID: PMC2660815. English.

31. Sieg A, Brenner H. Cost-saving analysis of screening colonoscopy in Germany. Zeitschrift fur Gastroenterologie. 2007 Sep;45(9):945-51. PubMed PMID: 17874356. English.

32. Sonnenberg A. Cost-effectiveness in the prevention of colorectal cancer. Gastroenterol Clin North Am. 2002 Dec;31(4):1069-91. PubMed PMID: 12489279. English.

33. Sorrentino D, Paduano R, Bernardis V, Piccolo A, Bartoli E. Colorectal cancer screening in Italy: feasibility and cost-effectiveness in a model area. Eur J Gastroenterol Hepatol. 1999 Jun;11(6):655-60. PubMed PMID: 10418938. English.

34. Subramanian S, Bobashev G, Morris RJ. When budgets are tight, there are better options than colonoscopies for colorectal cancer screening. Health Affairs. 2010 Sep;29(9):1734-40. PubMed PMID: 20671020. English.

35. Sweet A, Lee D, Gairy K, Phiri D, Reason T, Lock K. The impact of CT colonography for colorectal cancer screening on the UK NHS: costs, healthcare resources and health outcomes. Appl Health Econ Health Policy. 2011;9(1):51-64. PubMed PMID: 21174482. English.

36. van Ballegooijen M, Rutter CM, Knudsen AB, Zauber AG, Savarino JE, Lansdorp-Vogelaar I, et al. Clarifying differences in natural history between models of screening: the case of colorectal cancer. Med Decis Making. 2011 Jul-Aug;31(4):540-9. PubMed PMID: 21673187. English.

37. Ventura L, Zappa M, Carreras G, Ciatto S, Grazzini G. What is the best screening strategy to detect advanced colorectal adenomas? Simulation from ongoing Italian screening experiences. Tumori. 2011 Sep-Oct;97(5):547-50. PubMed PMID: 22158481. English.

38. Vijan S, Inadomi J, Hayward RA, Hofer TP, Fendrick AM. Projections of demand and capacity for colonoscopy related to increasing rates of colorectal cancer screening in the United States. Aliment Pharmacol Ther. 2004 Sep 1;20(5):507-15. PubMed PMID: 15339322. English.

39. Whynes DK. Cost-effectiveness of faecal occult blood screening for colorectal cancer: results of the Nottingham trial. Crit Rev Oncol Hematol. 1999 Nov;32(2):155-65. PubMed PMID: 10612015. English.

40. Whynes DK, Nottingham FOBST. Cost-effectiveness of screening for colorectal cancer: evidence from the Nottingham faecal occult blood trial. Journal of Medical Screening. 2004;11(1):11-5. PubMed PMID: 15006108. English.

41. Wohl P, Bednarik M, Wohl P, Cervenka M, Spicak J. Comparison of various strategies for colorectal cancer screening tests. Eur J Gastroenterol Hepatol. 2011 Nov;23(12):1157-64. PubMed PMID: 21989120. English.

42. Zauber AG, Lansdorp-Vogelaar I, Knudsen AB, Wilschut J, van Ballegooijen M, Kuntz KM. Evaluating test strategies for colorectal cancer screening: a decision analysis for the U.S. Preventive Services Task Force. Annals of Internal Medicine. 2008 4 Nov;149(9):659-69. PubMed PMID: 18838717. English.
