# Supplementary material for: Review of economic evidence in the prevention and early detection of colorectal cancer
Source: Health Econ Rev. 2013 Sep 12;3:20. doi: 10.1186/2191-1991-3-20 (PMC3847082; doi:10.1186/2191-1991-3-20)
Supplement: Additional file 4 — Summary of included studies. [file 2191-1991-3-20-S4.docx]

| **Country** | **Author (year)** | **Analytical perspective** | **Population  [yrs: years old]** | **Intervention(s) [q5Y: every 5 years]** | **Comparator(s)** | **Reported results [CE: cost-effective]** |
| --- | --- | --- | --- | --- | --- | --- |
| France | Chauvin (2012) | 3rd party payer | 50 - 80yrs average risk people | CTCq5Y CTCq10Y | immunological FOBTq2Y | Close expected net benefits between iFOBT and CTCq5 induced uncertainty in the choice of the optimal strategy. PSA then suggested that below a WTP per LYG of 8,587€/LYG, CTCq10Y was optimal, while CTCq5Y would be preferred beyond a WTP of 8,587€/LYG. |
| Singapore | Dan (2012) | Societal perspective | 50-75 yrs individuals | SIG once iFOBTq1Y BEq5Y COL once SIGq5Y SIGq5Y + iFOBTq1Y Stool DNAq5Y COLq10Y CTCq5Y iFOBT + COLq10Y | no screening | Performing single SIG at 60yrs was the cheapest screening strategy;Screening subjects 50 to 60 yrs old by iFOBT and subjects 60 to 72 yrs old with COL q10Y was the most CE strategy (USD $25,000/QALY). |
| US | Hassan (2010) | Societal | average risk 50yrs population in the US | NBI with resect and discard policy using (NBI (narrow band image)) q10Y | WL (white light)COLq10Y  no screening | With univeral referral of resected polyps to pathology, COL screening costs an estimated $3222/person, with a gain of 51 days/person |
| Canada | Heitman (2012) | Publicly funded healt care system | 50-75yrs age-two stratified groups (50-64yrs, 65-75yrs) Average risk population | FOBTq1Y (low- high-) FITq1Y(3 strategies-low, mid, high test performance) Fecal DNAq3Y FSIG q5Y CTC q5Y COLq10Y | No screening compared with each modality | FIT 1q1Y, assumng mid-range testing characteristics, was more effective and less costly compared to all strategies (including no screening) except FIT-high. Among the lifetimes of 100,000 average-risk patients, the number of CRC could be reduced from 4,857 to 1,782 and the number of CRC deaths from 1,393 to 457, while saving CAN$68 per person. |
| Canada | Heitman (2005) | To inform Canadian Health policy decision makers | 50 yrs | CTC then COL for adenomas 6mm or larger | COL | CTC for CRC screening would cost $2.27 m extra per 100 000 patients screened; 3.78 peroration-related deaths would be avoided, but 4.11 extra deaths would occur from missed adenomas. Because CTC screening would cost more and result in more deaths overall compared with COL, the latter remained the dominant strategy. Our resutls were sentivity to CTC test performance characteristics, the malignant risk of missed adanomas, the risk of perforation and related death, the procedural costs and differences in screening adherence. |
| France | Heresbach (2010b) | 3rd party payer only direct costs | Average risk 50-74yr, follow up model considered people with adenoma | gFOBT q2Y | iFOBT q2Y CTC q10Y | CTC requires substantially less COL than iFOBT and is CE for low values of WTP(less than 20000/LYG). However, iFOBT is the preferred screening strategy for a WTP greater than 6207euro/LYG. |
| Canada | Ho (2008) | publicly funded health care system | average-risk Canadians aged 50-74yrs; average risk is defiend as being asymptomatic with no personal or family history of adenomatrous polyps or CRC, or personal history of conditions that increase the risk of CRC. | CTCq10Y | COLq10  FOBTq1Y No screening | When compared to no screening using a cost-effectiveness framework, COL, FOBT, and CTC were associated with incremental gains in quality-adjusted life expectancy (0.04, 0.02, and 0.03 additional QALY, respectively). In the base case, the no screening strategy was the least expensive, followed by FOBT and then COL. CTC was associated with worse clinical outcomes and higher costs than COL. Compared with no screening COL was associated with a cost per QALY gained of $7,937. In PSA, compared with no screening, COL is associated with a cost per QALY of less than $10,000 nearly 99% of the time, while COL is cost saving in conmparison to CTC 91% of the time. |
| US | Khandker (2000) | payer's perspective | 50yrs and older without predisosing factors (screening 50-85yrs) | 8 strategies; 1)annual FOBT 2)FSIG q3Y 3)FSIG q5Y 4)annual FOBT and FSIG q5Y 5)DCBE q5Y  6) COL q5Y  7) COL q10Y | no screening | Lifetime costs of CRC $643 per eprson with no screening, compared to $2058 (annual FOBT), $2079 (FSIG q3Y), $1713 (FSIG q5Y), $2854 (FOBTannual and FSIG q3Y), $2639 (annual FOBT and FSIG q5Y), $2577 (DCBE q5Y), $3906 (COL5), $2602 (COL10) SIG q5Y and annual FOBT were the most cost-effective strategies, but with low compliance, FOBT was less cost-effective. |
| UK | Lee (2010) | NHS | Average risk 60-69yrs CRC screening | CTC | FOBT FSIG COL | CRC q10Y was CE compared with biennial FOBT. |
| France | Lejeune (2004) | French health-care insurance system | individuals until 85 | FOBT (Hemoccult-II test)q2Y 50-74yrs | no screening | Modelling biennial screening vs the absence of screening over a 20-year period resulted in a 17.7% mortality reduction and a discounted incremental cost-effectiveness ratio of €3,357 per life-years gained among individuals 50-74 years of age. Sensitivity analyses performed on epidemiological and economic data showed the strong impact on the results of colonoscopy cost, of compliance to screening, and of specificity of the screening test. |
| France | Lejeune (2010) | French health-care insurance system? | Individuals aged 50 to 74 (screening offered) and followed until 85yrs or deaths | Guiac nonrehydrated FOBT(gFOBT) q2Y | immunochemical FOBT(iFOBT)  no screening | Compared with no screening, gFOBT and iFOBT were associated with a decrease in colorectal cancer mortlaity of 17.4% and 25.2%, respectively. With regard to cost-effectiveness, expressed as cost per life-years gained, iFOBT was the most effective and most costly alternative. Compared with no screening, gFOBT and iFOBT presented similar discounted incremental cost-effectiveness ratios: €2739 and €2819 respectively per LYG. When compared with gFOBT, iFOBT presented an ICER of 2988 per LYG. |
| US | Loeve (1999) |  | 50yrs olds | **Planned** intervention(s) for economic evaluation: FSIGq3Y (till 74yrs) unhydrated FOBT q2Y (till 80yrs) | no screening | All kinds of assumptions on the natural history of CRC and screening and surveillance strategies can easily be incorporated in the model. MISCAN-COLON gives detailed output of incidence, prevalence and mortality, and the resutls and effects of screening. It can be used to test hypotheses about the natural history of colorectal cancer, such as the duration of progressive adenomas, and screening characteristics, such as sensitivity of test, against empirical data. |
| US | Loeve (2000) |  | 50yrs olds | FSIGq3Y (till 74yrs) unhydrated FOBT q2Y (till 80yrs) | no screening | Given the expert opinion-based assumptions, a program based on q5Y SIG screenings could result in a net savings of direct health care costs due to prevention of cancer treatment costs that compensate for the costs of screening, diagnostic follow-up, and surveillance. This resutl persists when costs and health effects are discounted at 3%. The 'break-even'point, the time required for a screening program that terminates after 30 years and 44 years for a screening program that continues on indefinitely. however, net savings increase assumptions about natural history of CRC, costs of screening, surveillance, and disgnostics are considered. |
| US | Lansdorp-Vogelaar (2009a) | not stated ?3rd party payer perspective | 50-80yrs general population | CTC with 20Y, 15Y, 10Y, 5Y intervals (1) intensive referral: any suspected polyps  (2)intermediate referral: suspected polyp>=6mm  (3) minimal referral: suspected poplyp >=10mm | COL q20Y, q15Y, q10Y, q5Y; | with equal costs ($662), COL dominated CTC screening. For CTC to gain similar LGY as COL q10Y, it should be offered q5Y with referral of polyps >=6mm |
| US | Lansdorp-Vogelaar (2010) | 3rd party payer | 65yrs olds (varied aged to 50 in SA) | Stool DNAq3Y Stool DNA q5Y | No screening HII HS iFOBT SIGB SIG HII + SIGB HII + SIG HS + SIGB HS + SIG HSq3Y + SIGB HSq3Y + SIG iFOBT + SIGB iFOBT + SIG iFOBTq3Y + SIGB iFOBTq3Y + SIG COL | Assuming a cost of $350 per test, strategies of stool DNA testing q3Y or q5Y yielded fewer LY and higher costs than the currently recommended CRC screening strategies. Screening with the stool DNA test would be CE at a per-test cost of $40 to $60 for stool DNA testing q3Y. depending on the simulation model used. There were no levels of sensitivity and specificity for which stool DNA testing would be cost-effective at its current cost of $350 per test. Stool DNA testing q3Y would be CE at a cost of $350 per test if the relative adherence to stool DNA testing were at least 50% better than that with other screening tests. |
| US | Eddy (1990) | not stated | 50yrs persons at average risk and persons at high risk because of having a 1st degree relative with CRC. | FOBT (Hemoccult-II)q1Y plus ACBEq3Y FOBTq1Y plus 60cm FSIGq5Y  FOBTq1Y plus COLq5Y FOBq1Y plus ACBEq1Y FOBTq1Y plus COLq3Y FOBTq1Y plus COLq1Y | no screening | Screening persons for 25 years, for the age of 50 to the age of 75yrs should reduce the chance of developing or dying from CRC by 10% to 75%, depending on which screening tests are used and how often screening is done. |
| US | McMahon (2001) | re-analysis of Eddy1990 Glick1998 OTA1995 |  | FOBT (Hemoccult-II)q1Y plus ACBEq3Y FOBTq1Y plus 60cm FSIGq5Y  FOBTq1Y plus COLq5Y FOBq1Y plus ACBEq1Y FOBTq1Y plus COLq3Y FOBTq1Y plus COLq1Y | no screening | Strategies in which DCBE examination was performed emerged as optimal from all studies included. In average-risk individuals, screening with DCBE examination q3Y, or q5Y with annual FOBT, had an ICER of less than $55,600 per life-years saved. Howeve,r DCBE examination screening q3Y plus annual FOBT had an ICER of more than $100,000 per LYS. COL had an ICER of more than $100,000 per LYS, was dominated by other screening strategies, and offered less benefit than did DCBE. |
| US | Ness (2000) | Societal perspective | 40 year old men and women in the US | One-time COL at; 45-49yrs 50-54 55-59 60-64 | no screening | We determined that one-time COL screening in men age, 60yr and in women age <65yr dominates never screening and screening at older ages. For both sexes, one-time COL screening between 50 and 54yrs is associated with a marginal cost-utility of less than $10,000 per QALY compared to screening between 55 and 60 yrs of age. One-time COL screening between 45 and 49ys of age is either dominated (women) or associated with a marginal cost-utility of $69,000/QALY (men) compared to screening between 50 and 54 yr of age. The marginal cost-utility of one-time COL screening is relatively insensitive to plausible changes in the cost of COL, the cost of CRC treatment, the sensitivity of COL for colorectal neoplasia, the utility values representing the morbidity associated with the CRC-related health states, and the discount rate. |
| US | Pickhardt (2008a) | not stated | 60yrs asymptomatic polyps; diminutive (<-5mm), small (6-9mm), large (>-10mm) | CTC then COL | CTC then No COL | Estimated 10Y CRC risk for unresected diminutive (0.08%), small (0.7%) and large polyps (15.7%). ICER of removing all diminutive $465,407, and small CTC-detected polyps $59,015 per LYG. Polypectomy for large CTC-detected polyps yielded a cost-saving of $151 per person screened. |
| US | Pickhardt (2008b) | not stated | 60yrs asymptomatic people with small polyps (6- to 9-mm) detected at CTC screening | 3Y CTC surveillance | Immediate polypectomy | Without any intervention, the estimated 5-year CRC death rate from 6- to 9-mm polyps in this concentrated cohort was 0.08%, which is a sevenfold decrease over the 0.56% CRC risk for the general unselected screening population. The death rate was further reduced to 0.03% with the CTC surveillance strategy and to 0.02% with immediate colonoscopy referral. However, for each additional cancer-related death prevented with immediate polypectomyversus CTC follow-up, 9,977 COL referrals would be needed, resulting in 10 additional perforations and an incremental CE ratio of $372,853. |
| US | Saini (2010) | not stated | 50yrs with newly diagnosed adenomas surveillance COL until 85yrs | COL q3Y HR, q10Y LR (3/10) 3/5 3/3 | No surveillance | 3/5 USD5743/QALY gained compared with no surveillance, 3/3 strategy was dominated by 3/5 strategy. Assuming that probability of advanced adenoma formation was 1.3% per year (based 0.5%), the ICER of the 3/5 strategy was <50000/QALY gained if the RR of advanced adenoma formaiton was <2.4 (base 3.9) |
| Ireland | Sharp (2012) | A healthcare payer perspective, Health Service Executive (HSE) | 55-74yrs | 1)gFOBYq2Y 55-74yrs, with reflex FIT 2)FIT q2Y 55-74yrs 3)FSIG once-only at 60yrs | no screening | All scenarios would be considered highly cost-effective compared with no screening. The lowest ICER vs no screening (ICER vs no screening €589 per QALY gained) was found for FSIG, followed by FIT (1696) and gFOBT (4428); gfFOBT was dominated. Compared with FSIG, FIT was associated with greater gains in QALYs and reductions in lifetime cancer incidence and mortality, but was more costly, required considerably more COL and resulted in more complications. |
| US | Song (2004) | not stated; "indirect costs were not included" & "aimed to explore the potential role of F-DNA in a national strategy" | 50-80yrs average risk of developing CRC | F-DNA q5Y COL q10Y FOBT q1Y FSIG q5Y FOBT + FSIG | No screening | Compared with no screening fDNA at a screening interval of 5Y decreased CRC incidence by 35% and CRC mortality by 54% and gained 4560 life-years per 100,000 persons at 47,700/LYG in the base case. However, fDNA gained fewer LY and was more costly than conventional screening. The average number of COL per person was 3.8 with COL strategy and 0.8 with fDNA strategy. In most 1-way SA and Monte Carlo simulation iterations, fDNA remained reasonably cost-effective compared with no screening, but COL and FOBT dominated fDNA. Assuming fDNA testing sensitivities of 65% for CRC and 40% for large polyp, and 95% specificity, a screening interval of 2Y and a test cost of $195 would be required to make fDNA comparable with COL. |
| US | Parekh (2008) | not stated |  | fecalDNA q3Y FOBTq1Y iFOBT COLq10Y |  | In the case, FOBT and iFOBT gained LY per person and cost less than no screening. Fecal DNA testing version 1.1 at $300 (the current PreGen Plus test) gained 5323 LY/100 000 persons at $16 900/LYG and fecal DNA testing version 2 (enhanced test) gained 5795 LY/100 000 persons at $15 700/LYG vs no screening. In the base case and most sensitivity analyses, FOBY and feacal immunochemocal testing were preferred to faecal DNA testing. Faecal DNA testing version 2 cost $100 000/LYG vs faecal immunochemical testing when per-cycle adherence with faecal immunochemical testing was 22%. Faecal immunochemical testing with excellence adherence was superior to COL every 10Y. |
| US | Ladabaum (2004) | National perspective | average risked US population 50yrs for 50 yrs time horizon | CTCq10Y (test performance reported by Cotton et al) CTCq10Y (base case) CTCq10Y( with test performance as reported by Pickhardt(2003)) | No screening COL | In the best case considered (95%, 94%, and 87% sensitivity for CRC, polyps>- 10mm, and polyps <10mm), CTC was nearly as effective as COL. However, if test costs were equal, total cost per person was 15% greater for CTC than COL, making COL dominant. When test cost for CTC was <-60% of test cost for COL, the small benefit of COL vs CTC cost >$200,000/incremental life-year. The greater the likelihood of being referred for COL after CTC, the greater the advantage of COL. with 75% screening adherence in the US, CTC and COl cuold decrease CRC incidence by 46%-54%, with COl requiring 6.9 million COL/year, and CTC, 3.2million COL/year, plus 5.4million CTC/year with CTC. |
| US | Ladabaum (2006) | not stated |  |  |  | As screening uptake increased, CRC incidence and mortality decreased, and annual costs related to CRC care and testing increased for younger persons, but decreased for older persons. Compared with current screening uptake of 40%, screening 75% of the US poulation aged 50 to 80 increased annual costs related to CRC care and testing from $3.5 billion to $5.0 billion for 50 6o 64 years olds, but decreased annual costs form $5.9 billion to $5.6 billion for those aged 65 years and older. Sensitivity analyses suggest that future costs for other diseases could offset CRC care savings in older Americans that are attributable to screening. However, wven without net cost savings for any age group, screening remained relatively cost-effective. |
| US | Sonnenberg (2000) | 3rd party payer | 50yrs olds general population, annual cycle till death | FOBTq1Y  FSIG q5Y COLq10Y | no screening | Compared with COL, FOBTq1Y costs less but saves fewer life-years. A screening strategy based on FSIG q5Y or 10Y is less cost-effective than the other two screening methods. |
| UK | Tappenden (2007) | National Health Service (NHS) UK | 50yrs and older | (1) FOBT biennial 50-69yrs (2) FOBT biennial 60-69yrs (3) FSIG once at 55yrs (4)FSIG once at 60yrs (5) FSIG once at 60yrs, followed by FOBT 61-70yrs | No screening | FSIG with or without FOBT (4,5) may be cost-saving and may produce additional benefits compared with no screening. The marginal cost-effectiveness of FOBT options compared to no screening is estimated to be below 3000GBP per QALY gained |
| US | Wagner (1991) |  | 65yrs old individuals until 85yrs or death | 1)FOBTq1Y and SIGq3Y 2)FOBTq2Y and SIGq5Y 3)FOBTq1Y and SIG at 65yrs 4)FOBT | No screening | A program of annual FOBT in the elderly would detect at least 17% of the expected cases of cancer and could cost $35 000 per year of life saved. Screening schedules that include peoridic SIG would prevent more cases of cancer but could cost between $43 000 and $47 000 per year of life gained. These estimates are based on uncertain assumptions, but results were not extremly sensitive to further relaxation of the values of the most uncertain assumptions. in no case did the cost per LYG from annual FOBT exceed %55 000 or did the cost per LYG from FOBT with SIG every 5yrs exceed %61 000. |
| US | Theuer (2006) | not stated | 50 yrs olds Black, Latinos, Asians and white men and women | Annual FOBT plus FSIGq5Y | COL q10Y | Age-specific CRC incidence rates were highest in balck men and lowest in Latino women. Screening beginning at age 50 was most cost-effective in balck men and least cost-effective in Latino women (measured in USD/LYS) using annual FOBT testing combined with FSIG q5Y and using COL q10Y. The cost-effectiveness of a 35-yrs screening program in black men beginning at age 45 was similar to the cost-effectiveness of screening white men and balck women beginning of age 50 and more cost-effective than screening nonbalck women as well as Asian and Latino men beginning at age 50. |
| US | Theuer (2001) | not stated | 50yrs olds Black, Latinos, Asians and white men and women | Annual FOBT plus SFIGq5Y | COL q10Y | Average annual age-specific CRC incidence rates were highest in blacks and lowest in Latinos. Screening beginning |
| US | Vijan (2001) | 3rd party payer (listed as one of limitations) | 50yrs | once- lifetime COL twice-lifetime COL | FOBT FOBT+FSIG FSIG COL | With 100% comp rate, twice-lifetime COL at 50yrs and 60yrs and FSIG with FOBT are most effective. Comp with primary screening tests and FU for polyps affect screening decisions. COL at 50 and 60yrs is the preferred test regardless of comp with the primary screening test. However, if FU COL for polyps is less than 75%, then even cone-lifetime COL is preferred over most combinations of FSIG and FOBT. |
| US | Maciosek (2006) | not stated | average-risk 50 yrs and older | FOBT q1Y FSIG q5Y COL q10Y |  | If a birth cohort of 4 million were offered screening at recommended intervals, 31,500 deaths would be prevented and 338,000 years of life would be gained over the lifetime of the birth cohort. In the current cross-section of people aged 50 and older, 18,800 deaths could be prevented each year by offering all people in this group screening at recommended intervals. Only 58% of these deaths are currently being prevented. In year 2000 dollars, the cost effectiveness of offering patients aged 50 and older a choice of colorectal cancer screening options is $11,900 per year of life gained. |
| US | Vijan (2007) | not stated | 50yrs till 80yrs (screening), modelled till 100yrs | 2DCTC q5Y+COL 2DCTC q10Y+COL 3DCTC q5Y+COL 3DCTC q10Y+COL | No screening current practice (FOBT, COL, FSIG) | COL dominates 2D CTC q5Y or q10Y. COL is weakly dominant over 3D CTC q5Y or q10Y. 3D CTC q5Y is more effective than COL q10Y, but costs an incremental $156000 per LYG. SA showed that 3D CTC q5Y is a dominant strategy of COL costs 1.6times more than CTC. COL is a dominant strategy if the sensitivity of CTC for 1cn adenomas is 83% or lower. |
| Australia | Walleser (2007) | Government perspective | individuals with a positive FOBT | CTC | COL | CTC is less effective and more costly than COL; if CTC was more sensitive than COL, CTC was more effective, at higher cost. |
| Taiwan | Wu (2006) | 3rd party payer perspective | general population 50-75y | Stool DNAq3Y Stool DNAq5Y Stool DNAq10Y | No screening FOBYq1Y FSIGq5Y COLq10Y | Stool DNA testing every three, five, and ten years can reduce CRC mortality by 22%, 15%, and 9%, respectively. The associated incremental costs were $9,794, $9,335, and $7,717, per LYS when compared with no screening. Stool DNA testing strategies were the least cost-effective with the cost per stool DNA test, referral rate with disgnostic COL, prevalence of large adenoma, and discount rate being the most influential parameters. |
| US | Zauber (2010) | CMS perspective on CTC | US population 65y-85y (50y in SA) | 14)CTC DoD 15)CTC ACRIN | 1)no screening 2)Hemoccult II(HII) 3)Hemoccult SENSA (HS) 4)FIT 5)SIGbiopsy  6)SIG  7)HII + SIGb 8)HII + SIG  9)HS + SIGb 10)HS + SIG 11)FIT + SIGb 12)FIT + SIG 13)COL | The CEA suggests that annual high sensitive FOBTs (guaiac and FIT), FSIGq5Y with sensitive FOBTq1Y, and COL are reasonably cost-effective strategies for CRC. Memoccult II only and FSIG only are not included in this set of acceptable tests. Similarly, with current levels of test costs based on diagnostic procedures, CTC is not a cost-effective choice. |
| US | Knudsen (2010) | Payer's perspective (CMS and modified societal excluding productivity cost) | average risked 65yrs old individuals | CTC DoDq5Y CTC NCTCq5Y | 1)H-IIq1Y 2)Hemoccult SENSA (HS) q1Y 3)iFOBTq1Y 4)SIGq5Y 5)SIGBiopsyq5Y 6) 1) + 4) 7) 1) + 5) 8) 2) + 4) 9) 2)+ 5) 10) 3) + 4) 11) 3) + 5)  12)COLq10Y 13)no screening | Assuming perfect adherence with all tests, the undiscounted number life-years gained from CTC screening ranged from 143 to 178 per 1000 65yrs olds, which was slightly less than the number of life-years gained from 10-yearly COL (152-185 per 1000 65 yrs-olds) and comparable to that from 5-yearly SIG with annual FOBT (149-177 per 1000 65yrs-olds). If CTC screening was reimbursed at $488 per scan (slightly less than the reimbursement for a COL without polypectomy), it would be the most costly strategy. CTC screening could be cost-effective at $108-$205 per scan, depending on the microsimulation model used. Sensitivity analyses showed that if relative adherence to CTC screening was 25% higher than adherence to other tests, it could be cost-effective if reimbursed at $488 per scan. |
| US | Vanness (2011) | US health sector perspective | average risk asymptomatic 50 yrs olds (screened till 80yrs & simulated till death) in the US | FOBTq1Y + FISGq5Y FITq1Y + FSIGq5Y COLq10Y CTqC5Y (5 mm referral threshold) CTCq10Y (5 mm referral threshold)  each strategy was run through CRC-SPIN, MISCAN, SimCRC models |  | CTC at 5- and 10-year intervals was more costly and less effective than FOBT plus FSIB in all three models in both 100% and 50% adherence scenarios. COL also was more costly and less effective than FOBT plus FSIG, except in the CRC-SPIN model assuming 100% adherence (ICER $26,300/LYG). CTC at 5- and 10-year screening intervals and COL were net beneficial over the 10-year interval except in the MISCAN model when assuming 100% adherence and WTP $50,000/LYG. |
| US | Subramanian (2009) | 3rd party payer, only direct medical costs | Average risk 50yrs, screening till 80yrs preference (pref) and compliance (comp) incorporated | FOBTq5Y FOBTq10Y FSIGq5Y FSGIq10Y COLq5Y COLq10Y comp at 45% (1) | no CRC screening (2) Scenario (3) 45% comp with 35% population never been screened Scenario (4) 100% comp | Improved comp is positively related to the reduction of CRC mortality. Achieving higher levels of compliance with screening or diagnosis recommendations such as targeted education and use of navigators to assist patients to increase adherence. |
| UK | Macafee (2008) | not specifically stated but top-down costs (direct costs) were considered | 60yrs olds, modelled for 50 yrs for two timescale: 2003 (early cohort) and 2033 (late cohort) | unhydrated FOBT q2Y 60-69yrs (2003 cohort) unhydrated FOBT q2Y 60-69yrs (2033 cohort) | no screening compared with corresponding cohort (early or late) | Life expectancy was assumed to increase by 2.5 years per decade. There were 407 552 fewer people entering the model in the 2033 model due to a lower birth cohort, and population screening saw 30 345 fewer CRC-related deaths over the 50 years of the model. Screening the 2033 cohort cost £96 million with cost savings of £43 million in terms of detection and treatment and 28 million GBP in palliative care costs. After 30 years of follow-up, the cost per life year saved was £1544. An identical screening programme in an early cohort (2003) saw a cost per life year saved of £1651. |
| Canada | Telford (2010) | 3rd party paper | 50yrs average risk screening and surveillnce till 75yrs olds | gFOBT low Se q2Y gFOBT low Se q1Y gFOBT high Se 1Y gFOBT low Se q1Y plus SIG q5Y FIT q1Y Fecal DNA q1Y DCBE q5Y CTC q5Y COL q10Y | NO screening | current strategies reduced CRC incidence and mortality compared with no screening ICER CAD $9159 (gFOBT low Se q1Y), CAD $611(FIT q1Y), CAD $6133 (COL q10Y) |
| Italy | Di Bidino (2010) | HC system perspective | average risk | Arm 1 FOBT or FSIG or BE then COL for all +ve results /COL/ CTC vs | Arm 2 FOBT Arm3 FSIG Arm4 BE Arm5 COL Arm6 CTC | Arms 3,4, and 6 showed strong dominance compared with Arm1, ICERs of Arm2 and Arm5 below threshold value of €35,000.  Technology overlapping is not cost-effective |
| US | Sonnenberg (2002) | 3rd party payer perspective | 50 yrs olds hypothetical population | COL once at 65yrs COLq10Y from 50yrs of age | No screening | Comapred with no screening, the ICER of a single or repeated COL amounts to $2981 or to $10983 per LYG, respectively. A single COL saves most life years if done at the age of 60, but becomes most CE after the age of 70. Depending on the level of compliance, repeated COL save 2-3 times more lives than a screening program based on a single COL. |
| US | Howard (2005) | not stated | 50 yrs olds who are offered annual FOBT and follows them until death | FOBT | No screening same as Sonneberg 2000 | The way in which participation rate is modelled, particularly assumptions made about the subsequent screening behaviour of non-participants (‘íf' and 'when' a non-participant attends for subsequent screening), affects the cost-effectiveness estimates for FOBT screening programmes. 100% participation in all screening gives USD/LYS of $9705.Under |
| US | Pickhardt (2007) | not stated | people 50yrs with small (6-9mm) polyps detected at CTC screening | CTC with no polyp size reporting threshold CTC with a 6-mm polyp size reporting threshold | COL plus popypectomy FSIG  No screening | Compared with No screening; $4361(CTC with a 6-mm threshold), $7138 (CTC with no threshold), $7407(FSIG), $9180 (COL). Compared with COL, CTC with a 6-mm threshold resulted in a 77.6% reduction in invasive endoscopic procedures and 1112 fewer reported COL-related complications from perforation or bleeding. |
| Italy | Hassan (2007) | National level in Italy | 50yrs for 30 yrs | All q10Y; CTC screening  COL FS | No screening | 65% initial adherence and a compliance with rpt examinatios of 80%, COLq10Y appeared to be the most effective technique preventing 40.9% of CRC, whilst CTC resulted to be less effective than COL (38.2%), but more effective than FSIG (31.8%), corresponding to 3821, 3589, and 2945 LYS, respectively. |
| US | Hassan (2008) | Societal | 50yrs and over | CapEndo q10Y (capsule endocsopy; Pillcam Colon) | COL q10Y no screening | At baseline, the incremental cost-effectiveness (compared with no screening) of COL and CapEndo was $16,165 and $29,244 per LYS, respectively. When equal compliance was simulated, the COL program was more effective and less costly than a strategy based on CapEndo. When simulating an initial compliance to CapEndo 30% better than COL, CapEndo became more effective and more cost-effective option. A 20% better compliance was sufficient when a higher accuracy of CapEndo for polyps was assumed. A 6 mm threshold for polypectomy referral was associated with a substantial cost reduction in the CapEndo program with only a small loss of efficacy. |
| US | Hassan (2009b) | Societal perspective | hypothetical cohort aged 50-100 yrs | COLq10Y CTC q5Y FSIG q5Y BE q5Y | not stated See Hassan (2008) CE capsule endoscopy | In the reference-case analysis, COL was optimal test with the highest NB ($1945 per subject invited for screening compared with $1862, $1717, and $1653 for CTC, FISG, and BE, respectively). Results of PSA indicated that COL was the optimal choice in only 45% of the simulated scenarios, whereas CTC, FSIG, and BE were the optimal strategies in 23%, 16%, and 15% of the scenarios, respectively. Only two parameters were responsible for most of this uncertainty about the optimal test for CRC screening: the increase in adherence with less invasive tests and CRC natural history. The expected societal monetary benefit of further research in these areas was estimated to be more than $15 billion. |
| Canada | Flanagan (2003) | 5% | hypothetical sample of 7,001,322 people 50-74yrs with no history of CRC | FOBTq2Y (Hemoccult II nonrehydrated) | no screening | Compared with no screening, the discounted ICER of biennial screening was $11,907. The ICNER of annual screening was $13,497.  When the costs were increased, the ICER was $18,445 with biennial screening and $19,893 with annual screening. Participation rate was an important determinant of the CE of the screening programme. When the participation rate was reduced from 67% to 50%, the biennial screening became less cost effective ($15,688). |
| US | Frazier (2000) | Perspective not clearly stated (costs include the actual costs of medical personnel and supplies to provide the services AND overhead costs) | 50yrs average risk (screening surveillance till 85yrs) | rFOBT(rehydrated) uFOBT(unrehydrated) FSIG DCBE COL | No screening | FOBT rehydrated (annual) + FSIG plus COL (if polyp found) ICER USD92900/LYG compared with no screening among white men |
| US | Hassan (2009a) | Societal | 60yrs postpolypectomy surveillance | at 1Y COL surveillance postpolypectomy | no referral for COL after polypectomy | "COL at1Y as compared with a no COL at 1Y postpolypectomy, was a relatively CE with an ICER of $66,136, whici is well below the artibrary threshold of $150,000" |
| US | Helm (2000) | not stated, possibly societal perspective | 45-74 yrs general population in the US | FOBT | No screening | More than 1 million CRC could be expected to arise over 10 yr in the cohort of US residents eligible to enter a screening program in 1997, and trial outcomes indicate that ≥60% of these cancers would be fatal. If the 60-67% compliance rate of the population-based RCTs were achieved, a FOBT program would detect 30% of known CRCs and save 100,000 lives over 10 yr. Screening would incur total costs of $3-4 billion over 10 yr, or $2,500 per life-year saved. |
| US | Heresbach (2010a) | 3rd party payer | CTC offered at 50yrs, 60yrs, 70yrs | CTC without polyp size reporting threshold (PL strategy) CTC with polyp size reporting threshold (TS) | No screening | ICER of PL and TS strategies were 12042 and 2765/LYG associated to CRC prevention rates of 37.9 and 36.5%. ICER of PL and TS strategies dropped to 9687 and 1857/LYG when AA prevalence increased from 6.9 to 8.6% for male participants and 3.804.9% for female participants or to 9482 and 2067/LYG when adenoma and AA annual recurrence rates dropped to 3.2 and 0.25%. ICER for PL and TS strategies decreased to 7947 and 954/LYG or when only two CTC performed at 50 and 60yrs. conversely ICEr did not significanlty change when varying population rate or accuracy of CTC. |
| US | Sonnenberg (1999) | 3rd party payer | 50yrs average risk | CTC q10Y MRC (magnetic resonance colonography) | polypectomy then; COL q10Y COL q3Y | Under baseline conditions, screening by COL $20,930 per LYS, CTC $24,586 per LYS. ICER comparing CTC to no screening and COL to CTC were $11,484 and $10,408, respectively. Col screening remains more CE even if the Se and Sp of CTC both rise to 100%. For the two screening procedures to become similarly CE, CTC needs to be associated with an initial compliance rate 15-20% better or procedural costs 54% less than COL. |
| Australia | Stone (2004) | 3rd party payer | Australian population at average risk | CRC FOBTq2Y 55-69yrs olds | Current practice (opportunistic screening) Base program extension to include; 45-49 50-54 70-74 75+ | We estimate a minumum of 'base program'o fscreening those aged 56 to 69 years could avert 250 deaths per annum (95% uncertainty interval 99-440), at a gross cost of $A55 million (95% UI $A46 million to $A 96 million) and a gross incremental cost effectiveness ratio of $A 17,000/DALY (95% UI $A 13,000/DALY to $A 52,000/DALY). Extending the program to include 70 to 74 yeas olds is a more effective option (cheaper and higher health gain) than including the 50 to 54 yrs olds. |
| USA | Regge (2009) | Societal perspective (indirect costs for CTC and COL have been estimated, given a median hourly income rate of $18.62) | Average risk 50yrs | CTC with CAD (software, computer aided detection)  CTC without CAD as a 2nd reader performed by radiologists with different level of experience. | CTC FSIG COL No screening | CAD CTC vs CTC, $8661/LYG (inexperience readers), $61354/LYG (experienced readers); COL vs CAD CTC $498 668/LYG (experienced). For inexperienced readers CAD CTC was more clinically effective and CE than FSIG |
| Singapore | Wong (2004) | not stated | 50-70 yrs of age, time horizon of 50Y | gFOBT q1Y iFOBT q1Y DCBE q3Y FSIG q5Y COL q10Y | No screening | "Results are reported by giving the average cost and life expectancy for the subgroup within the population from the age of 50-69 through to 70." FOBT is superior in terms of cost for LIS, SGD 162.11/LYS at 100% compliance. COL was most expensive strategy. |
| China | Tsoi (2008) | ? Not stated Payer perspective | 50yrs average risk | FOBT FOBT then COL FSIG q5Y COL then CS q3Y for polyps COL then FOBT q10Y for no-polyps | No screening . | Assume comp rate 90%, ICER for FOBT USD 6222/LYS and COL USD 7211/LYS. Even comp rate of FOBT were 50% and 30%, FOBT has the lowest ICER. |
| US | Lansdorp-Vogelaar (2009b) | not stated | 40yrs old balck and white men and women in the US | COLq10Y COLq8Y individualized COL according to gender and race (white men 53-74yrs COLq7Y, black men 47-75yrs COLq7yrs, white women 53-77yrs COLq8Y, black women 47-75yrs COLq7Y) | no screening | The base case strategy of no screening was the least expensive, yet least effective. The uniform 10Y COL strategy was dominated. The uniform 8Y COL and individualized strategies both increased life-expectancy by 0.0433-0.0435 years per individual at a cost of $15,565 per LYG. In the individualized strategy, African Americans began screening 6years earlier with a 1year shorter interval compared with whites. The individualized policies were essentially the same for men and women, because the higher CRC risk in men is offset by their shorter life-expectancy. The results were robust for changes in model assumptions. |
| Australia | O'Leary (2004) | government-funded health system | 50-64yrs, analysis over a 10-year time frame | FSIG q10Y COL q10Y FOBT q1Y FOBT q2Y | No screening | Col averted the greatest No of cases of CRC (35%), followed by FSIG *25%), and annual (25%) and biennial (14%) FOBT. COL averted the greatest number of deaths from CRC (31%), followed by annual FOBT (28%), FSIG (21%) and biennial FOBT (19%). FSIG was the most efficient in terms of cost per LYS (A$ 16,801), followed by COL (A$ 19,285), biennial (A$ 41,183), and annual (A$ 46,900) FOBT. |
| Korea | Park (2005) | Korean NHI | 50yrs average risk in NHI | COLq5Y COLq3Y COLq10Y COLat50 SIGq3Y SIGq5Y SIGq10Y SIGat55 SIGq5Y+DCBEq5Y FOBTq2Y FOBTq1Y DCBEq10Y DCBEq5Y DCBEq3Y | no screening | With the NHI did not cover the screening and compliance was 30%, non-dominated strategies were COLq5Y and COLq3Y. In all scenarios of various compliance rates with raised coverage of the NHI and increased reimbursement of OCL, COLq10Y, COLq5Y and COLq3Y were non-dominated strategies, and COLq10Y had lower or minimal incremental medical cost and financial burden on the NHI than the strategy of no screening. These results were stable with sensitivity analyses. |
| France | Sobhani (2011) | payer - health care system | people without symptoms | iFOBTq2Y MagStream 1xsample  gFOBTq2Y OC-SENSOR 1xsample OC-SEONSOR 2xsample OC-SENSOR 3xsample |  | The results suggest that a 3-sample iFOBT with 50 ng/mL as a positive cut off is cost-effective. It provides more asymptomatic cancer detection without significantly increasing normal COL |
| The Netherlands | van Rossum (2011) | 3rd party payer | 50-75yrs Dutch population time horizon 10 years | iFOBT once gFOBT once | no screening | iFOBT dominated the alternatives: after one round of iFOBT screening, a hypothetical persone would on average gain 0.003 life-years and save the health care system 27 Euro compared with gFOBT and 0.003 LY and 72 Euro compared with no screening. Overall, in 4,460,265 Dutch aged 50-75yrs, after one round iFOBT screening, 13,400 LY and 320 million Euro would have been saved compared with no screening. iFOBT also dominated in sensitivity analyses, varying uncertainty surrounding important effect and cost parameters. |
| China | Wang (2012) | not stated | 50-80yrs Chinese individuals | Repeat COL | single COL no screening | Assuming a first-time compliance rate of 90%, repeat screening COL and single COL can reduce the incidence of CRC by 65.8% and 67.2% respectively. The incremental cost-effectiveness ratio for single COL (49 Renminbi Yuan[RMB]) was much lower than that for repeat screening COL (474 RMB). Single COL was a more cost-effective strategy, which was not sensitivity to the compliance rate of COL and the cost of advanced CRC. |
| UK | Whyte (2012) | NHS | 50-100yrs | gFOBT at 60-69 q2Y gFOBT at 60-74 q2Y iFOBT at 60, 65, 70yrs iFOBT at 60-69yrs iFOBT at 60-74yrs q2Y FSIG age 55yrs FSIG 55, 65yrs FSIG 55yrs and gFOBT q2Y 66-74yrs FSIG 55yrs and iFOBT 60,65,70 FISG 55yrs, and iFOBT 60-74 q2Y FSIG 55yrs, and iFOBT 56-74 q2Y | no screening | The model suggests that screening strategies involving FSIG or iFOBT (immunochemical FOBT) may produce additional benefits compared with the current policy of FOBTq2Y for 60-74yrs. The age at which a single FSIG screen results in the greatest QALY gain was 55, with similar gains for ages between 52 and 58. Strategies which combined FSIG and iFOBT showed further benefits and improved economic outcomes. |
| The Nethelands | Wilschut (2011a) | not stated | Age to start screening (45,50,55,60), stop screening age (70,75,80) in Netherlands (N=30000) | Different FIT cut off level of 50, 75, 100, 150, 200 ng/mL Hb. For each cut off level, screening strategies were assessed with various age ranges and screen intervals | see intervention(s) | At all cost levels, FIT screening between age 55 and 75 yrs using FIT at 50 ng/mL, for example, was €3900/LYG. FIT screening is more cost-effective at a cut off level of 50 ng/mL than at higher cut off levels - which is considerably lower than the values used in current practice. |
| The Netherlands | Wilschut (2011b) | not stated | 45-80yrs Dutrch population - attendance rate, costs, positivity, and detection rates from two Dutch implementation trials were analysed | FIT at varying Hb cut off levels under different capacities | gFOBT | When COL capacity was unlimited, the optimal screening strategy was to administer an annual FIT with a 50 ng/mL Hb cutoff level in individuals aged 45-80yrs and to offer COL surveillance to all individuals with adenomas. When Col capacity was decreasing, the optimal screening adaptation was to first increase the FIT Hb cutoff value to 200 ng Hb per mL and narrow the age range ot 50-75yrs, to restrict COL surveillance, and finally to further decrease the number of screening rounds. FIT screening was always more cost-effective compared with gFIBT. Doubling COL capacity increased the benefits of FIT screening up to 100%. |
